# Postoperative morbidity of complete mesocolic excision and central vascular ligation in right colectomy: a retrospective comparative cohort study

**DOI:** 10.1186/s12957-018-1514-3

**Published:** 2018-10-30

**Authors:** Gian Andrea Prevost, Manfred Odermatt, Markus Furrer, Peter Villiger

**Affiliations:** 1 Department of Surgery, Kantonsspital Graubünden, Loëstrasse 170, CH-7000 Chur, Switzerland; 2 Private University of the Principality of Liechtenstein, Triesen, Principality of Liechtenstein

**Keywords:** Right colectomy, Complete mesocolic excision, Central vascular ligation, Morbidity, Mortality

## Abstract

**Background:**

To investigate morbidity and mortality following complete mesocolic excision (CME) and central vascular ligation (CVL) in patients undergoing right colectomy.

**Methods:**

Data from consecutive patients undergoing elective right colectomy at a university-affiliated referral centre were retrospectively analysed. Patients who underwent conventional right-sided colonic cancer surgery (January 2001–April 2009, *n* = 84) were compared to patients who underwent CME/CVL (May 2009–January 2015, *n* = 71). The primary end point was anastomotic leak. Secondary end points were delayed gastric emptying, severe respiratory failure, mortality and length of hospital stay.

**Results:**

No significant difference was found in the rate of anastomotic leak (1.2% in the conventional versus 5.6% in the CME/CVL group, *p* = 0.108). Patients in the CME/CVL group had a higher 90-day mortality rate (7.0% versus 0.0%, *p* = 0.019). Four out of five deceased patients suffered from aspiration with consecutive respiratory failure. There was a tendency towards delayed gastric emptying in the CME/CVL group (12.7% versus 7.1%, *p* = 0.246). Clavien-Dindo complication grades ≥ 2 were similar in both groups with 16 (19%) in the conventional and 15 (21.1%) in the CME/CVL group (*p* = 0.747). CME/CVL patients had a shorter mean length of stay with 11 versus 14 days (*p* <  0.001).

**Conclusions:**

Complete mesocolic excision with central vascular ligation in right colectomy seems to have a higher aspiration rate leading to severe respiratory failure and to higher mortality compared to conventional resection methods. Patient selection for this procedure may therefore be crucial.

## Background

Total mesorectal excision (TME) is a well-established technique for the management of rectal cancer which has significantly reduced local recurrence rate [1]. In recent years, the concept of complete mesocolic excision (CME) with dissection adhering to embryological planes and central vascular ligation (CVL) has also been adopted to colonic resection [2–6]. While data exist describing an increased disease-free survival in patients who have undergone right colectomy using CME [7], little is known about the perioperative morbidity and mortality associated with CME/CVL in the specific anatomical proximity of pancreas and duodenum [8–11]. Although the CME/CVL procedure has proven to be feasible and may even prolong disease-free survival, in our experience, a specific array of postoperative problems seems to occur more commonly in CME/CVL than in conventional right colectomy. In particular, delayed gastric emptying with consecutive pulmonary aspiration and anastomotic leaks seem to have increased since the introduction of CME/CVL. Due to the lack of randomised controlled trials confirming an increased overall survival of CME/CVL for right colectomy, it is questionable whether the potentially increased complication rate outweighs the probable oncological benefits of this method.

We therefore conducted a single-centre retrospective cohort study to determine whether there is an increase in morbidity associated with CME/CVL compared to conventional right colectomy.

## Material and methods

### Patients and methods

**Fig. 1:**
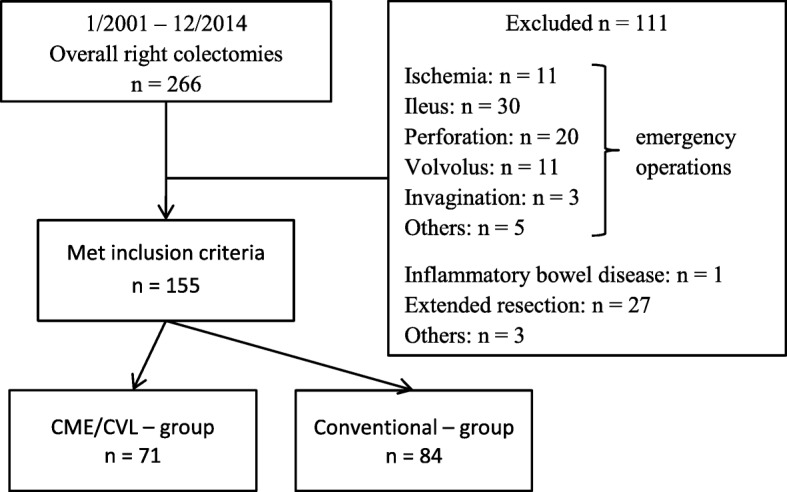
Selection of patients for the study

From January 2001 to December 2014, a total of 266 patients underwent right colectomy in our university-affiliated referral hospital. Only adult patients (> 18 years) who underwent elective right colectomy for confirmed or suspected primary malignant tumours were included as shown in Fig. 1. Exclusion criteria were multi-visceral resections, concomitant inflammatory bowel disease and patients denying consent for analysis of their personal data. A total of 155 patients met the inclusion criteria whose characteristics are shown in Table 1. Patients with suspected malignant tumours were operated in the same technique as patients with confirmed malignancies. Therefore, short-term outcome should not differ significantly between those groups allowing for inclusion of both groups for final analysis.

**Table 1:** Baseline parameters

	Conventional group	CME/CVL group	*p* value
	*n* = 84	*n* = 71	
Patient characteristics			
Female gender	42 (50.0)	32 (45.1)	0.540
Age (years)*	73.8 (21.0–88.9)	73.6 (33.0–88.9)	0.843
Arterial hypertension	34 (38.6)	38 (52.1)	0.088
Coronary artery disease	10 (11.9)	12 (16.9)	0.374
Cerebrovascular insult	0 (0.0)	5 (7.0)	0.019
Diabetes mellitus	5 (6.1)	13 (18.3)	0.019
Chronic kidney disease	4 (4.8)	7 (9.9)	0.218
Dialysis	0 (0.0)	2 (2.8)	0.208
Chronic obstructive pulmonary disease	5 (6.0)	4 (5.6)	1.000
Body mass index**	24.9 (4.3) (16.6–34.9)	25.5 (3.7) (18.1–35.6)	0.361
Smoker	16 (19.0)	9 (12.7)	0.283
Alcohol abuse	9 (10.7)	3 (4.2)	0.132
Steroids	2 (2.4)	2 (2.8)	1.000
Previous abdominal surgery	46 (54.8)	41 (57.7)	0.709
ASA 1	3 (3.6)	2 (2.8)	
ASA 2	56 (66.7)	48 (67.6)	
ASA 3	25 (29.8)	21 (29.6)	0.964
Perioperative parameters			
Operation time (minutes)**	155 (52) (62–315)	174 (48) (105–374)	0.020
Anaesthesia time (minutes)**	279 (67) (140–510)	292 (52) (204–465)	0.179
First surgeon: consultant	35 (41.7)	47 (66.2)	0.002
Laparoscopic operation	0 (0.0)	22 (31.0)	< 0.001
Laparoscopy-assisted operation	10 (11.9)	28 (39.4)	< 0.001
Extended right colectomy	6 (7.1)	5 (7.0)	0.981
Stapler anastomosis	57 (67.9)	70 (98.6)	< 0.001
Side-to-side anastomosis	57 (67.9)	69 (97.2)	< 0.001
Insertion of drains intraoperatively	45 (53.6)	49 (69.0)	0.050
Intraoperative fluid balance (ml)**	2642 (1021) (500–5800)	1558 (803) (180–4890)	< 0.001
Continuous epidural analgesia	65 (77.4)	62 (87.3)	0.109
Preoperative bowel preparation	26 (31.0)	2 (2.8)	< 0.001

With percentages in parentheses unless indicated otherwise

*ASA* American Society of Anesthesiologists classification

*Values are median (range)

**Values are mean (standard deviation) (range)

The CME/CVL method was implemented in May 2009 after observerships in institutions already using this method, video tutorials and practical workshops. In the later period of conventionally performed resections, laparoscopy has increasingly been adopted and became the primary approach. Patients being in the conventional control group had surgery from January 2001 to May 2009 whilst the CME/CVL group underwent surgery from May 2009 to January 2015.

The tested alternative hypothesis was that CME/CVL has higher perioperative morbidity than conventional resection. The primary end point was anastomotic leak. Secondary end points were delayed gastric emptying, severe respiratory failure, mortality and length of hospital stay.

Ninety-day institutional mortality was reported because Byrne et al. [12] showed that extending mortality reporting to 90 days identifies a greater number of operation and hospitalisation-associated deaths when compared to the 30-day period. Delayed gastric emptying was defined as nasogastric tube removal after postoperative day 3. Severe respiratory failure was defined as required intubation or non-invasive treatment with continuous positive airway pressure. Chronic kidney disease was defined as a glomerular filtration rate of < 45 ml/min/1.73 m^2^ at admission, estimating the glomerular filtration rate with the CKD-EPI (Chronic Kidney Disease Epidemiology Collaboration) equation [13].

### Procedures

#### Conventional resection

After lateral to medial mobilisation of the right colon, division of the transverse colon and the terminal ileum by electrocautery followed. The mesocolon was dissected in a V-shape manner towards the origin of the ileocolic and right colic vessels which were ligated. The anastomoses were mainly fashioned in a hand-sewn end-to-end technique. In the few cases of conventional procedures performed laparoscopically, the same technique as in the corresponding open operation was used. Resection of the exteriorised bowel as well as the anastomosis was performed via a transverse supraumbilical incision where a wound protector was applied.

#### Complete mesocolic excision and central vascular ligation

CMEs with CVL were usually performed laparoscopically with open procedures limited to selected patients with bulky tumours. In contrast to the technique of CME/CVL described by Hohenberger et al. [14], Kocherisation of the duodenum to harvest the retro-pancreatic central lymph nodes was not performed routinely.

In open CME/CVL, mobilisation of the right colon started laterally and continued centrally between the mesocolic surface layer and Gerota’s fascia. The ileocolic, the right colic, and the right branch of the middle colic vessels were divided at their origin. In extended right colectomies, the middle colic vessels were divided centrally at the level of the superior mesenteric artery and vein preserving the ileocolic trunk if present. Lymph node clearance around the central vessels and the superior mesenteric vein was performed. The greater omentum was divided at the resection level of the transverse colon and detached from the stomach. An isoperistaltic side-to-side stapler anastomosis was performed in most cases.

In contrast to the open resection, a medial to lateral mobilisation was performed in laparoscopic CME/CVL. The dissection started medial of the ileocolic vessels with creating a window in the mesentery. Following the ileocolic pedicle, the superior mesenteric vein was identified and cleared from lymphatic tissue up to and including the ileocolic trunk. The ileocolic as well as the right branch of the middle colic vessels were ligated and divided centrally. The medial to lateral mobilisation exposed the duodenum and head of the pancreas. Lateral division completed the mobilisation of the right colon. In complete laparoscopic procedures, the bowel was divided with endoscopic linear staplers and the specimen retrieved over a small suprapubic transverse laparotomy using a wound retractor. Finally, a laparoscopic side-to-side stapler anastomosis was performed. For laparoscopy-assisted procedures, a transverse supraumbilical incision was made wide enough to exteriorise and resect the mobilised bowel and to fashion the anastomosis by stapler.

### Assessment of the specimen

From May 2009 onwards, specimens were examined adhering to the grading system used for the MRC CLASSIC trial complemented with the subsequently introduced fourth category [15]. In this grading system, specimens were classified as follows:

- Grade 1/“poor”: moderate bulk of mesocolon and disruptions extending down onto the muscularis propria
- Grade 2/“moderate”: moderate bulk of mesocolon, disruptions not reaching down onto the muscularis propria
- Grade 3/“good”: intact mesocolon and smooth peritoneal-lined surface
- Grade 4: pathologist’s classification as grade 3 and surgeon reports central dissection

### Perioperative management

The patient was admitted the day before surgery. Bowel preparation was a routine procedure in the conventional group only. Single-shot antibiotic prophylaxis with Cefazolin and Metronidazole was administered in both groups. Abdominal drains and nasogastric tubes were inserted routinely. Nasogastric tubes were removed either immediately after the operation or on the following day in cases with low tube output. The time of drain removal was decided by the primary surgeon. Liquids and solid food were administered as soon as tolerated. There was no change in postoperative nutrition policy over the study period. In particular, there was no enhanced recovery program established for both groups.

### Data analysis

Data were collected from clinical records and pooled in an electronic database. Statistical analysis was performed using SPSS version 18 (SPSS Inc., an IBM Company Chicago, Illinois, USA). Discrete variables were compared with the chi-square test or Fisher exact test, as appropriate. Means of continuous data were compared using the Student’s *t* test for normally distributed data and the Mann-Whitney *U* test for not normally distributed data. Normality was determined graphically using histograms. *P* values ≤ 0.05 were considered to be significant.

## Results

Included for analysis were 155 patients with 84 patients in the conventional group and 71 patients in the CME/CVL group. Baseline parameters of the two groups are shown in Table 1. Characteristics of the malignant tumours are listed in Table 2.

**Table 2:** Malignant tumour characteristics

	Conventional group	CME/CVL group	*p* value
	*n* = 71 (84.5)	59 (83.1)	0.810
Tumour locations			
Ileocoecal (ileum, appendix, coecum)	29 (40.8)	34 (57.6)	
Ascending colon	29 (40.8)	16 (27.1)	
Right flexure and transverse colon	13 (18.3)	9 (15.3)	0.149
T-stage			
Tumour in situ (Tis)	1 (1.4)	1 (1.7)	
T1	5 (7.0)	5 (8.5)	
T2	7 (9.9)	9 (15.3)	
T3	51 (71.8)	36 (61.0)	
T4	7 (9.9)	8 (13.6)	0.771
N-stage			
N0	38 (53.5)	41 (69.5)	
N1	18 (25.4)	8 (13.6)	
N2	15 (21.1)	10 (16.9)	0.143
M-stage			
M0	60 (84.5)	55 (93.2)	
M1	9 (12.7)	4 (6.8)	
Mx	2 (2.8)	0 (0.0)	0.217

With percentages in parentheses

The primary end point, namely anastomotic leak, was reached in only one case (1.2%) in the conventional group versus four cases (5.6%) in the CME/CVL group, the difference not being statistically significant (*p* = 0.180). However, a significant difference was found in the postoperative 90 day institutional mortality rate with zero cases in the conventional group and five cases (7.0%) in the CME/CVL group (*p* = 0.019). The first patient, a 78-year-old man with an adenocarcinoma of the ascending colon, died on postoperative day 5 most likely from aspiration caused by repeated vomiting. The second patient, an 89-year-old man with a cecal adenocarcinoma died on postoperative day 8 in the intensive care unit from respiratory insufficiency after aspiration and consecutive pneumonia. The third patient, an 82-year-old female patient with a metastasised adenocarcinoma of the caecum, died on the 53rd postoperative day due to prolonged gastroparesis and consecutive aspiration. The fourth patient, a 74-year-old man with a large adenoma of the ascending colon, died on the 7th postoperative day from a cardiovascular arrest after fulminant aspiration. A septic shock caused by an anastomotic insufficiency led to a consecutive abdominal compartment syndrome requiring emergency laparotomy and an ileostomy on postoperative day 7 where the patient died the same day because of cardiac organ failure. The fifth patient, a 77-year-old man with an adenocarcinoma of the transverse colon, died on postoperative day 19 from a pulmonary embolism after an operative revision of an anastomotic leak on postoperative day 12. All four patients with aspiration had a nasogastric tube reinserted because of nausea and vomiting. Overall however, the frequency of complications Clavien-Dindo ≥ 2 was not significantly different in the two groups with 16 (19%) in the conventional and 15 (21.1%) in the CME/CVL group (*p* = 0.747).

Intra- and postoperative complications are listed in Table 3.

**Table 3:** Complications

	Conventional group	CME/CVL group	*p* value
	*n* = 84	*n* = 71	
Intraoperative apparent complications	12 (14.3)	14 (19.7)	0.367
Vascular injuries	4 (4.8)	7 (9.9)	0.218
Blood loss (ml)*	300 (50–4000)	100 (40–800)	< 0.001
Post-operative complications			
Clavien-Dindo ≥ 2	16 (19)	15 (21.1)	0.747
Surgical site infections			
Superficial and deep incisional	13 (15.5)	8 (11.3)	0.446
Organ/space	3 (3.6)	4 (5.6)	0.703
Anastomotic leak	1 (1.2)	4 (5.6)	0.180
Iatrogenic small bowel perforation	1 (1.2)	1 (1.4)	1.000
Bleeding	4 (4.8)	2 (2.8)	0.688
Sepsis	0 (0.0)	2 (2.8)	0.208
Pneumonia	3 (3.6)	4 (5.6)	0.703
Severe respiratory failure	0 (0.0)	5 (7.0)	0.019
Pulmonary embolism	1 (1.2)	2 (2.8)	0.593
Cardiac decompensation or atrial fibrillation	1 (1.2)	3 (4.2)	0.333
Acute renal insufficiency	0 (0.0)	3 (4.2)	0.094
Urinary tract infection	4 (4.8)	1 (1.4)	0.376
Urinary retention	4 (4.8)	0 (0.0)	0.125
90-day institutional mortality rate	0 (0.0)	5 (7.0)	0.019

With percentages in parentheses unless indicated otherwise

*Values are median (range)

The mean harvested lymph node count was 23.3 (SD 12.5) in the conventional and 32.2 (SD 17.4) in the CME/CVL group (*p* = 0.001). For the resection quality analysis, there were 12 cases missing. The median of the specimen resection quality in the CME/CVL group was 3. Twenty-nine (49.2% of the analysed specimen) had a resection quality of 4, 15 (25.4%) had a resection quality of 3, 14 (23.7%) of 2 and 1 (1.7%) of 1.

Details about the postoperative course are summed up in Table 4.

**Table 4:** Postoperative course

	Conventional group	CME/CVL group	*p* value
	*n* = 84	*n* = 71	
Duration of hospital stay (d)*	14 (8–43)	11 (6–35)	< 0.001
ICU postoperative	3 (3.6)	4 (5.6)	0.703
Reintervention	3 (3.6)	8 (11.3)	0.063
Needed antibiotic therapy	14 (16.7)	11 (15.5)	0.843
Time to first mobilisation (pod)**	1.10 (0.51) (0–4)	1.13 (0.58) (0–3)	0.722
Time to first flatus (pod)**	2.62 (1.13) (1–6)	2.04 (1.06) (1–6)	0.005
Time to first bowel movement (pod)**	4.17 (1.86) (1–11)	3.37 (1.61) (1–8)	0.005
Time to normal diet (pod)*	7 (4–26)	5 (1–18)	< 0.001
Delayed gastric emptying	6 (7.1)	9 (12.7)	0.246
Total length of stay of NGT (d)*	2 (1–12)	4.5 (1–14)	0.207
Urine catheter removal (pod)**	4.64 (3.02) (1–21)	3.27 (1.71) (0–9)	0.001
Drain removal (pod)*	5 (1–20)	3 (1–15)	0.002
Maximal weight gain (kg)**	4.47 (2.92) (−2–11)	3.78 (2.85) (− 2–11)	0.138
Total days with CEA*	5 (0–12)	4 (0–9)	0.029

With percentages in parentheses unless indicated otherwise

*ICU* intensive care unit, *pod* postoperative day, *d* days, *CEA* continuous epidural analgesia, *NGT* nasogastric tube

*Values are median (range)

**Values are mean (standard deviation) (range)

## Discussion

Our study shows a higher mortality in the CME/CVL group with aspiration and consecutive respiratory failure as the leading cause. Previous studies comparing CME/CVL to conventional right colectomies regarding perioperative morbidity are limited. Bertelsen et al. [8] revealed a higher postoperative morbidity in CME/CVL patients with a higher rate of intraoperative injury including splenic and superior mesenteric vein injuries and a higher rate of postoperative sepsis. Consistent to our data, Bertelsen et al. also found a higher respiratory failure rate in this collective compared to the conventionally operated group (8.1% versus 3.4%, *p* <  0.001). In contrast, a case series by Prochazka et al. [11] of 63 patients in a conventional group versus 20 patients in a CME/CVL group showed no difference in morbidity. Although in our study, the mortality rate of 7% seems to be unacceptably high, similar mortality rates for CME/CVL of the right colon have been reported in the past [16]. Our seemingly high mortality rate may be explained by the extended time period of 90 days defining institutional mortality. Furthermore, all of the deceased patients were of advanced age (median 78, range 74–89) and had substantial comorbidities (80% ASA 3).

Aspiration with respiratory failure proved to be the main cause of death (4 out of 5 patients) in CME/CVL patients. There was also a tendency towards longer nasogastric tube drainage in this group indicating prolonged postoperative gastroparesis as a risk factor for aspiration. This may be explained by the extensive mobilisation of the mesenteric root at the duodenal knee and pancreas head specific to CME/CVL right colectomy.

Two out of the five deceased patients suffered from anastomotic leakage, and one of them had a fulminant aspiration. Anastomotic leakage in colorectal surgery is a leading factor for postoperative morbidity. Bowel paralysis and gastroparesis are well-known disorders secondary to anastomotic leakage, but compared to other resection areas, right colectomy seems to have a negative impact on gastric emptying even in otherwise uneventful courses.

Increased rates of vascular injury, though not statistically significant, occurred in the CME/CVL group which may be caused by the more extensive dissection along the superior mesenteric vein. On the other hand, median intraoperative blood loss was significantly higher in the conventional group most likely because of the higher rate of open procedures in this group. Increased blood loss has been proven to adversely impact the operative outcome especially regarding anastomotic leakage [17]. Although lymphatic leaks are to be expected due to the more extensive lymphadenectomy, we did not experience isolated lymphatic leaks being clinically obvious and needing any specific treatment. However, we did not systematically screen for asymptomatic lymphoceles and some complications as abscesses or prolonged ileus might have been caused by lymphatic leaks in the first place.

Compared to the conventional group, a significant higher amount of procedures have been performed by a consultant in the CME/CVL group (66.2% versus 41.7%, *p* = 0.002). This was primarily due to the new and more demanding operation technique.

The main limitation of our study is its retrospective nature, the long observation period associated with changes in perioperative and adjuvant management and the lack of power because of small sample size. Though some of the differences in short-term outcomes seem to be clinically relevant, they did not reach statistical significance which may solely be due to small sample sizes.

The differences of operative and postoperative management in the two groups due to changing methods and modified managements in the last two decades may explain some of the differing results. Additionally, the whole learning curve of CME/CVL right colectomy, in particular laparoscopic CME/CVL, is included in the consecutive cases potentially increasing the complication rate in the CME/CVL group. Multiple previous studies have shown significant advantages of laparoscopic compared to open procedures including perioperative morbidity [18–22] which is in contrast to our results where more complications in the CME/CVL group occurred where most procedures were performed laparoscopically. Likewise did other factors like less intraoperative blood loss, better intraoperative fluid balance and less postoperative weight gain in the CME/CVL group not translate in a better short-term outcome. On the other hand, the prolonged gastroparesis in this group may support our hypothesis, namely that CME/CVL for right colectomy adversely affects gastrointestinal function postoperatively. Though ASA did not differ between the two groups, some risk factors may have increased the risk for complications in the CME/CVL group. In particular, diabetes mellitus and cerebrovascular disease rates were more frequently reported in the CME/CVL group and may partially explain the worse short-term outcome in this group. Diabetes mellitus as a known risk factor for delayed gastric emptying may have increased this effect in the CME/CVL group [23]. On the other hand, the abovementioned differences in baseline morbidities might be due to underreporting in the historic control group. At that time, Switzerland had a reimbursement system mainly focusing on the principal diagnosis so that comorbidity reporting in health records was not as rigorous as it became in later periods. This explanation may be supported by the fact that for example chronic kidney disease was reported significantly more commonly in the CME/VLE group although the average glomerular filtration rate at admission was similar in both group. Though not decreasing overall morbidity, the higher rate of laparoscopic procedures may explain the shorter hospital stay in the CME/CVL group. Also the aforementioned implementation of a new reimbursement system in Switzerland with Diagnoses Related Groups (SwissDRG) in January 2012 and its economic impact on health care might be a reason for shorter hospital stays [24].

## Conclusions

Taking into account the lack of randomised controlled trials and that current data [7] only strongly suggest a survival benefit for patients operated with the CME/CVL standard, careful patient selection to avoid the increased morbidity of the clearly more extensive procedure may be crucial especially in elderly patients. While mesocolic excision undisputedly has to be considered the standard of tumour surgery, the benefit of central vascular ligation and extensive lymph node clearing beyond the level of vessel ligation as a rigid oncologic principle to be applied in all patients remains unclear. As recent data [7] has been unable to show clear superiority of CME/CVL in regard to disease-free survival, an adequately powered randomised controlled trial is in our opinion ethically justifiable to determine the real long-term impact of CVL with extensive lymphatic tissue clearance as an independent factor besides newer chemotherapeutic regimens.

## Abbreviations

ASA: American Society of Anesthesiologists classification
CEA: Continuous epidural analgesia
CME: Complete mesocolic excision
CVL: Central vascular ligation
ICU: Intensive care unit
NGT: Nasogastric tube
Pod: Postoperative day
TME: Total mesorectal excision
